# CircRNA ITCH Inhibits Epithelial–Mesenchymal Transformation and Promotes Apoptosis in Papillary Thyroid Carcinoma via miR-106a-5p/JAZF1 Axis

**DOI:** 10.1007/s10528-024-10672-1

**Published:** 2024-02-15

**Authors:** Yijun Chen, Zhiming Lian, Guolie Zhang, Yuanmei Lin, Guoliang Zhang, Wei Liu, Jian Gao, Zifang Zheng

**Affiliations:** First Department of Thyroid Surgery, The Affiliated Hospital of Putian University, No. 999 Dongzhen East Road, Licheng District, Putian, 351100 Fujian China

**Keywords:** Papillary thyroid carcinoma, Circ-ITCH, EMT, miR-106a-5p, JAZF1

## Abstract

Circular RNA ITCH (circ-ITCH) is implicated in papillary thyroid carcinoma (PTC) development. Nevertheless, the more detailed molecular mechanism remains uncovered. The transcriptional level of circ-ITCH was tested via quantitative real-time PCR. Transwell assay was introduced to assess the migrative and invasive abilities of cells. RNA interference technology was employed to reduce the level of circ-ITCH as well as JAZF1 in PTC cells. Western blot assay was utilized to reveal the content of JAZF1 and proteins related to epithelial–mesenchymal transformation (EMT) progression. Circ-ITCH was downregulated in PTC tissues as well as cells. Overexpression of circ-ITCH suppressed EMT, migration, invasion, facilitated apoptosis in PTC cells, while silencing circ-ITCH exhibited reversed effects. Additionally, miR-106a-5p was the target of circ-ITCH and negatively regulated through circ-ITCH. MiR-106a-5p mimic partly eliminated the influences of overexpressed circ-ITCH in PTC cells. Moreover, JAZF1 could interact with miR-106a-5p, then it was regulated via circ-ITCH. Silencing JAZF1 partially counteracted the role of circ-ITCH in PTC cells progress. This study uncovered that circ-ITCH suppressed the development of PTC cells at least partly by mediating miR-106a-5p/JAZF1 network.

## Introduction

Thyroid cancer is the most frequent malignancy of the endocrine system, and its incidence has been on the rise in recent years, increasing by more than 5% per year (Regenstein et al. 2016; Lim et al. 2017; Chen et al. 2009; Garg et al. 2014, 2017). Papillary thyroid carcinoma (PTC) is identified as the most frequent thyroid cancer (Schneider and Chen 2013). Currently, some traditional treatment alternatives such as surgery as well as radiation therapy have effectively improved the survival rate of PTC patients (Frohlich and Wahl 2014). Most sufferers with PTC will be successfully treated by these series of means, while a portion of PTC patients still develop complications and eventually die after treatment.

Circular RNAs (circRNAs), a recently discovered class of noncoding RNAs, are discovered to exhibit vital roles in diverse biological activities including carcinogenesis (Memczak et al. 2013; Zhou et al. 2018). For example, circRNA WHSC1 facilitates endometrial cancer progression by regulating miR-646/NPM1 axis (Liu et al. 2020). CircRNA ITCH (circ-ITCH) was firstly introduced by Memczak’s team. It is found that circ-ITCH serves as a suppressor in cancers (Yang et al. 2018; Ren et al. 2019; Zhao et al. 2021). Additionally, Emerging evidence proved circ-ITCH blocked PTC progress via miR-22-3p/CBL/β-catenin axis (Wang et al. 2018). However, more investigations should be conducted to enrich the molecular network of circ-ITCH in PTC progression and to more strongly verify whether circ-ITCH is able to be regarded as the therapeutic target of PTC.

MicroRNAs (miRNAs) as another vital endogenous non-coding RNAs consist of 19–24 nucleotides and play roles by degrading mRNAs or suppressing their transcription (Cheng et al. 2015; Martello et al. 2010; Kloosterman and Plasterk 2006). Increasing evidence illustrated that miRNAs are involved in multiple malignancies (Ma et al. 2020; Zheng et al. 2019; Wang et al. 2021). The starBase database proved that miR‐106a‐5p has potential sites that bind to circ-ITCH. Moreover, it is reported that miR-106-5p is associated with a variety of cancers including lung adenocarcinoma (Liu et al. 2021) and oral squamous cell carcinoma (Zhu et al. 2020). Nevertheless, it is unclear whether circ-ITCH exerts its roles in PTC cells via miR-106a-5p.

JAZF1 was first reported as a protein capable of interacting with TAK1 in 2004 (Nakajima et al. 2004). It is discovered to regulate diverse cellular functions like cell differentiation and inflammatory response (Johnson et al. 2018; Meng et al. 2018). Previous studies illustrated that JAZF1 regulates the development of diverse tumors, such as gastric cancer (Lim et al. 2017) and prostate cancer (Chen et al. 2009).

Besides, Huang et.al verified that JAZF1 inhibits growth and accelerates apoptosis of PTC cells (Huang et al. 2019). However, the upstream genes mediating JAZF1 in PTC cells remain uncovered. Interestingly, accumulating evidence proved that JAZF1 exerts a non-negligible role in cellular activities as the downstream target of diverse miRNAs (Lim et al. 2017; Garg et al. 2014). Previous results in the present study demonstrated that miR-106a-5p could inhibit the expression of its target gene, JAZF1 in PTC cells, which also aroused our great research interest.

This study revealed circ-ITCH weakened EMT progression and accelerated apoptosis in PTC cells through miR-106a-5p-mediated JAZF1 expression.

## Materials and Methods

### Samples

PTC tissues as well as para cancer tissues were collected from 8 patients with PTC in the Affiliated Hospital of Putian University, which was approved by the Ethics Committee of Affiliated Hospital of Putian University (Approval No. 2023082). Every patient in the study signed an informed consent form.

### Cell Culture

TPC-1, BCPAP, as well as Nthy-ori 3-1 cell lines were brought from Tongpai (Shanghai) Biotechnology Co., Ltd (China). IHH-4 cell line was brought from Shanghai Tongwei Industrial Co., Ltd (China). The cells were maintained in RPMI-1640 medium (R8758, Sigma-Aldrich, USA) in a 37 °C incubator with 5% CO_2_.

### QRT-PCR

Total RNA was collected from PTC tissues, para cancer tissues, or cells using TRIzol (T9424, Sigma-Aldrich, USA). Besides, the NE-PER Nuclear and Cytoplasmic Extraction Reagents (78833, Thermo Scientific, USA) were introduced to isolate the fractions in nuclear and cytoplasm of PTC cells. A Titan One Tube RT-PCR kit (11855476001, Roche, Switzerland) was employed to produce complementary DNA (cDNA) abiding by the manufacturer’s guidelines. The transcription levels of specific genes were detected by qPCR utilizing FastStart Universal SYBR Green premix (FSUSGMMRO, Roche, Switzerland) as described in the kit’s protocol. The qPCR was conducted as described in previous study (Peng et al. 2017). In simple terms, the reaction system is prepared as exhibited in Table 1 and the qPCR was performed on the ABI 7300 Real-Time PCR System (Applied Biosystems, USA) according to the procedure presented in Table 2. 2^−ΔΔCt^ method was introduced to estimate the level of target gene. GAPDH and U6 was regarded as the internal control for mRNAs, circRNA, and miRNAs. The sequence of all primers is exhibited in Table 3.

**Table 1:** The reaction system

Reagent	Volume (μl)
2 × SYBR green mix	10
cDNA	6.8
Primer forward (5 μM)	0.8
Primer reverse (5 μM)	0.8
H_2_O	1.6
Total	20

**Table 2:** The qPCR procedure

Temperature (°C)	Cycles	Time
50	1	2 min
95	1	10 min
95	40	15 s
60	40	1 min

**Table 3:** The primers of qRT-PCR

Gene	Primer
Circ-ITCH	Forward: 5′-AGCAATGCAGCAGTTT-3′
	Reverse: 5′-TGTAGCCCATCAAGACA-3′
Linear ITCH	Forward: 5′-GGAGACAACGCCTTAACC-3′
	Reverse: 5′-CATCTACTGTGACCTCTACG-3′
JAZF1	Forward: 5′-TGTAGCACCATGACAGGCATC-3′
	Reverse: 5′-TTGTCCTCGATGTGCTCGAT-3′
GAPDH	Forward: 5′-GGA GCGAGATCCCTCCAAAAT-3′
	Reverse: 5′-GGCTGTTGTCATACTTCTCATGG-3′
MiR-106a-5p	Forward: 5′-GGTAGGTCGTATCCAGTGCAA-3′
	Reverse: 5′-CGTATCCAGTGCGTGTCGT-3′
U6	Forward: 5′-CTCGCTTCGGCAGCACA-3′
	Reverse: 5′-AACGCTTCACGAATTTGCG-3′

### Transwell Assay

The migration or invasion of PTC cells were detected using Matrigel-free or Matrigel (354262, Corning, USA)-covered Corning HTS Transwell chambers (CLS3396, Corning, USA), as described in previous studies (Tang et al. 2021; Shang et al. 2020). Briefly, transfected PTC cells were collected in FBS-free RPMI-1640 medium and implanted in the upper chamber. The RPMI-1640 medium containing FBS was added to the lower chamber and then the cells were cultured at 37 °C for 24 h. Next, the PTC cells in upper chamber were removed. The cells in lower chamber were fixed with 4% polyformaldehyde fixing solution (60536ES60, Yesen, China) and stained with 0.1% crystal violet (60505ES25, Yesen, China). The migrated or invasive cells were recorded utilizing a microscope.

### Western Blotting

Tissues as well as cells were lysed using RIPA Lysis Buffer Suit (BI-WB015, SenBeiJia China). Then SDS-PAGE was introduced to isolate total protein and proteins were shifted to polyvinylidene fluoride membrane (MS-5020, Membrane Solutions, USA). Next, the membranes were incubated utilizing primary antibodies as shown in Table 4 and then treated with secondary antibodies. Finally, the protein was visualized with a kit (34095, Thermo Scientific, USA).

**Table 4:** The information of antibodies

Antibody	Item	Supplier	Country	Dilution
E-cadherin	20874-1-AP	Proteintech	USA	1:1000
N-cadherin	22018-1-AP	Proteintech	USA	1:1000
Vimentin	5741	CST	USA	1:1000
Snail	3879	CST	USA	1:1000
JAZF1	ab80329	Abcam	UK	1:1000
GAPDH	5174	CST	USA	1:1000

### Cell Apoptosis

An Annexin V-FITC-PI kit (APOAF, Sigma-Aldrich, USA) was employed to detect cell apoptotic rate according to the kit’s protocol. Briefly, transfected PTC cells (1 × 10^5^ cells/well) were harvested in binding solution containing 5 μl Annexin V-FITC as well as 5 μl Prodium Iodide. Then the mixture was incubated in the dark at 37 °C for 15 min. Finally, the apoptotic rate was recorded using a flow cytometer.

### Cell Transfection

MiR-106a-5p mimic (5′-AAAAGUGCUUACAGUGCAGGUAG-3′), miR-106a-5p inhibitor (5′-CUACCUGCACUGUAAGCACUUUU-3′), and miR-NC (5′-UUCUCCGAACGUGUCACGUTT-3′) were purchased from Genomeditech (China) and transfected into cells employing a reagent (L3000015, Invitrogen, USA) abiding by the manufacturer’s protocol. After 36–72 h of transfection, subsequent analysis was conducted.

For circ-ITCH, vectors with siRNAs targeted circ-ITCH (si-circ-ITCH-1#-5′-GUCCUUCAUAAUGAGCUUCAG-3′; si-circ-ITCH-2#-5′-ACCUGGAUGGGUUGAAGAATT-3′; si-circ-ITCH-3#-5′-AUGGGUUGAAGAAGUAGUUTT-3′) or JAZF1 (5′-UCUGUGACCAUUCUUAGCGUG-3′) and control vector (5′-UUCUCCGAACGUGUCACGUTT-3′) were obtained from GenePharma (China), and transfected into cells employing a reagent (L3000015, Invitrogen, USA) as described above.

### Luciferase Activity

PTC cells were co-transfected using vectors carrying with 3′UTR of circ-ITCH (wild type or mutant sequence) or JAZF1 (wild type or mutant sequence) and miR-106a-5p mimic, miR-106a-5p inhibitor or miR-NC. After 36–72 h, the supernatant was removed, and the activities of firefly and Renilla luciferase were measured with a Dual Luciferase Reporter Gene Assay Kit (RG027, Beyotime, China) according to the manufacturer’s protocol. The values were detected by employing a SpectraMax iD5- Multifunctional microplate reader (Molecular Device, China).

### Statistical Analysis

All data analyses used *p* < 0.05 to represent the level of significance. All analyses were conducted using GraphPad Prism 9.2 (USA). The paired *t* test as well as one-way ANOVA were introduced as appropriate.

## Results

### Circ-ITCH is Downregulated in Human PTC Tissues and PTC Cells than Normal Thyroid Cells

**Fig. 1:**
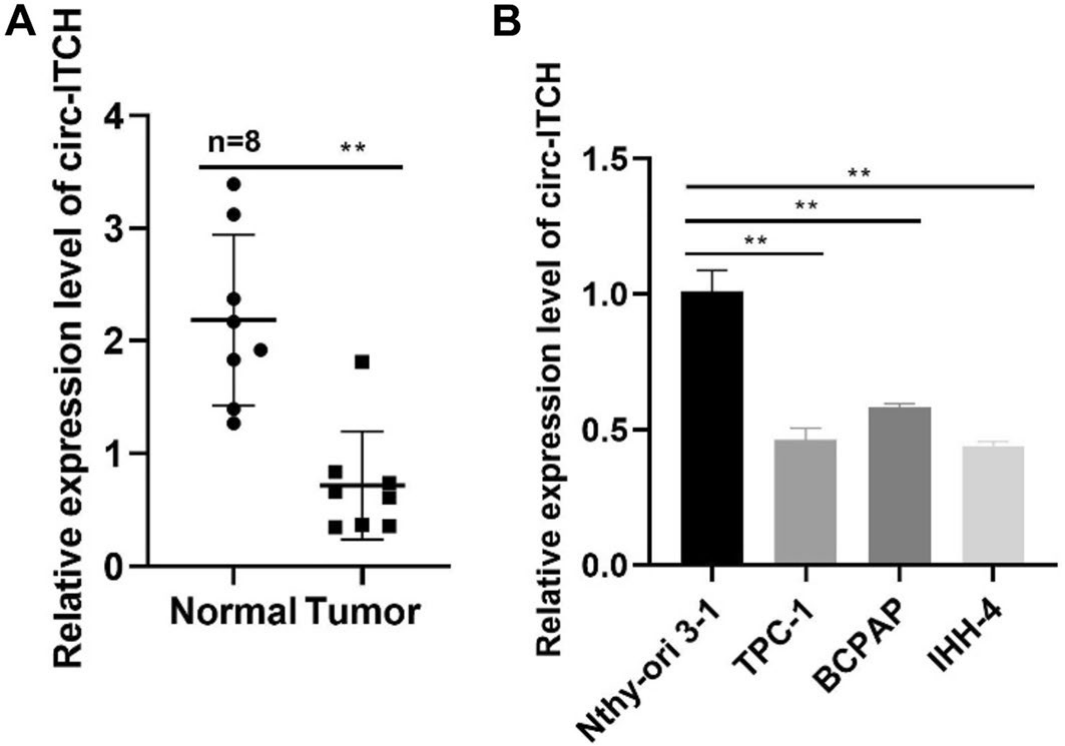
Circ-ITCH is downregulated in human PTC tissues and PTC cells than normal thyroid cells. **A** Circ-ITCH expression in tissues (*p* < 0.05). **B** Circ-ITCH expression in cells (*p* < 0.05)

PTC tissues and para cancer tissues (*n* = 8) were collected to estimate the expression level of circ-ITCH. The results demonstrated circ-ITCH was decreased in PTC tissues significantly (Fig. 1A). Moreover, circ-ITCH was reduced in PTC cells compared to normal human thyroid cells (Fig. 1B).

### Circ-ITCH Overexpression Inhibits PTC Cells Progression

**Fig. 2:**
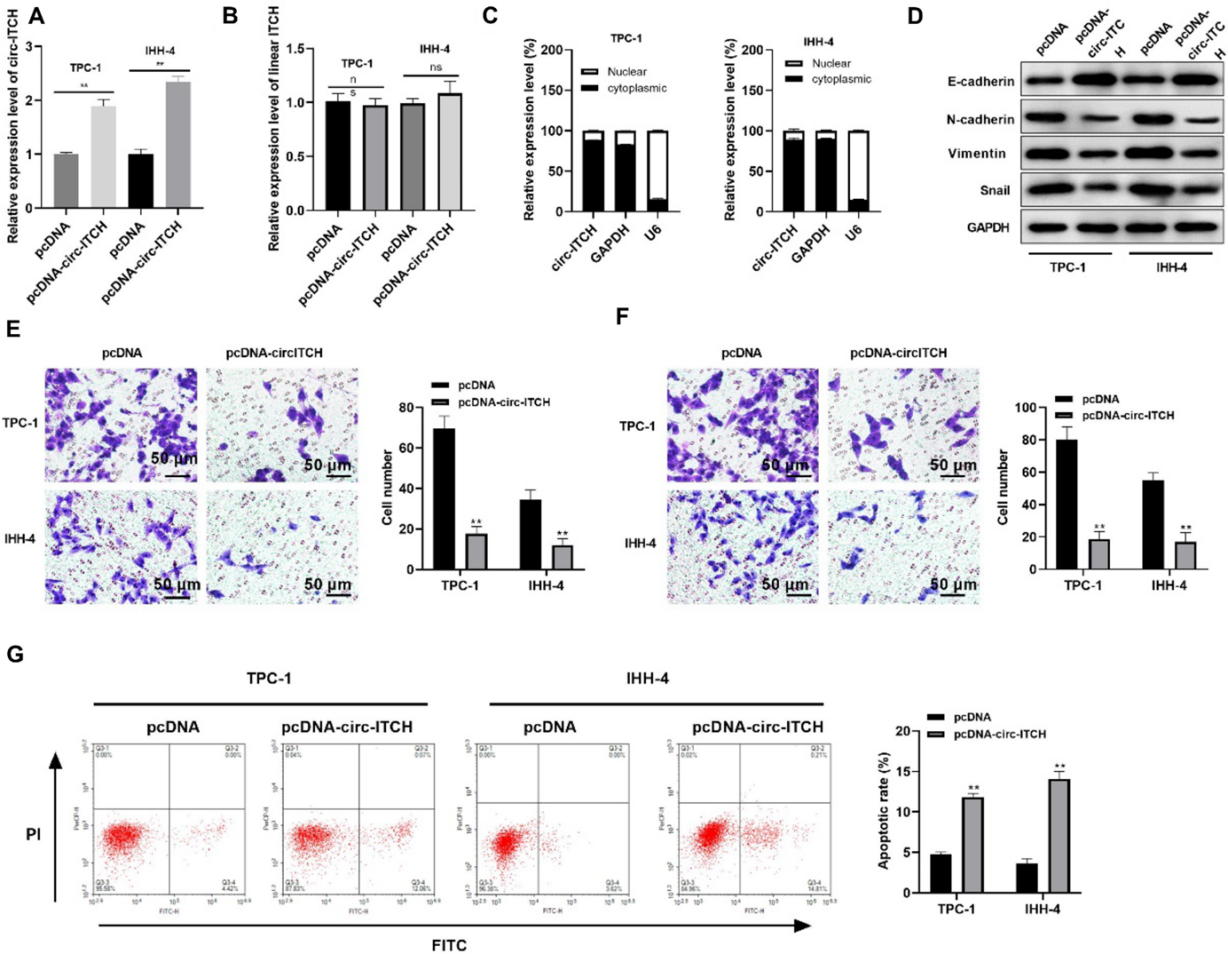
Circ-ITCH overexpression inhibited progression in PTC cells. **A** The mRNA level of circ-ITCH in cells (*p* < 0.05). **B** The mRNA level of linear ITCH in cells (*ns* not significant). **C** The localization of overexpressed circ-ITCH in cells. **D** The level of proteins related to EMT in cells. **E** and **F** The migration (**E**) and invasion (**F**) tested via transwell assay (*p* < 0.05). **G** The apoptosis rate tested via flow cytometry (*p* < 0.05)

Circ-ITCH was overexpressed in TPC-1 as well as IHH-4 cell lines by transfecting with circ-ITCH overexpressed plasmid (Fig. 2A). Besides, further assay proved that the linear ITCH mRNA did not change significantly (Fig. 2B), indicating that only circ-ITCH was indeed overexpressed. The qRT-PCR analysis illustrated that overexpressed circ-ITCH was mainly localized in cytoplasm rather than nucleus of TPC-1 as well as IHH-4 cells transfected with circ-ITCH plasmid (Fig. 2C). The subsequent experiments revealed that circ-ITCH overexpression upregulated the protein level of E-cadherin while reduced that of N-cadherin, Vimentin, as well as Snail, indicating that circ-ITCH blocked EMT progression of PTC cells (Fig. 2D). Moreover, increased circ-ITCH inhibited invasion in addition to migration of PTC cells remarkably (Fig. 2E, F). Furthermore, flow cytometry assay confirmed that overexpression of circ-ITCH facilitated apoptosis in TPC-1 as well as IHH-4 cells (Fig. 2G). Collectively, overexpressed circ-ITCH inhibited EMT, invasion, as well as migration, and accelerated apoptosis in PTC cells.

### Circ-ITCH Knockdown Promotes PTC Cells Progression

**Fig. 3:**
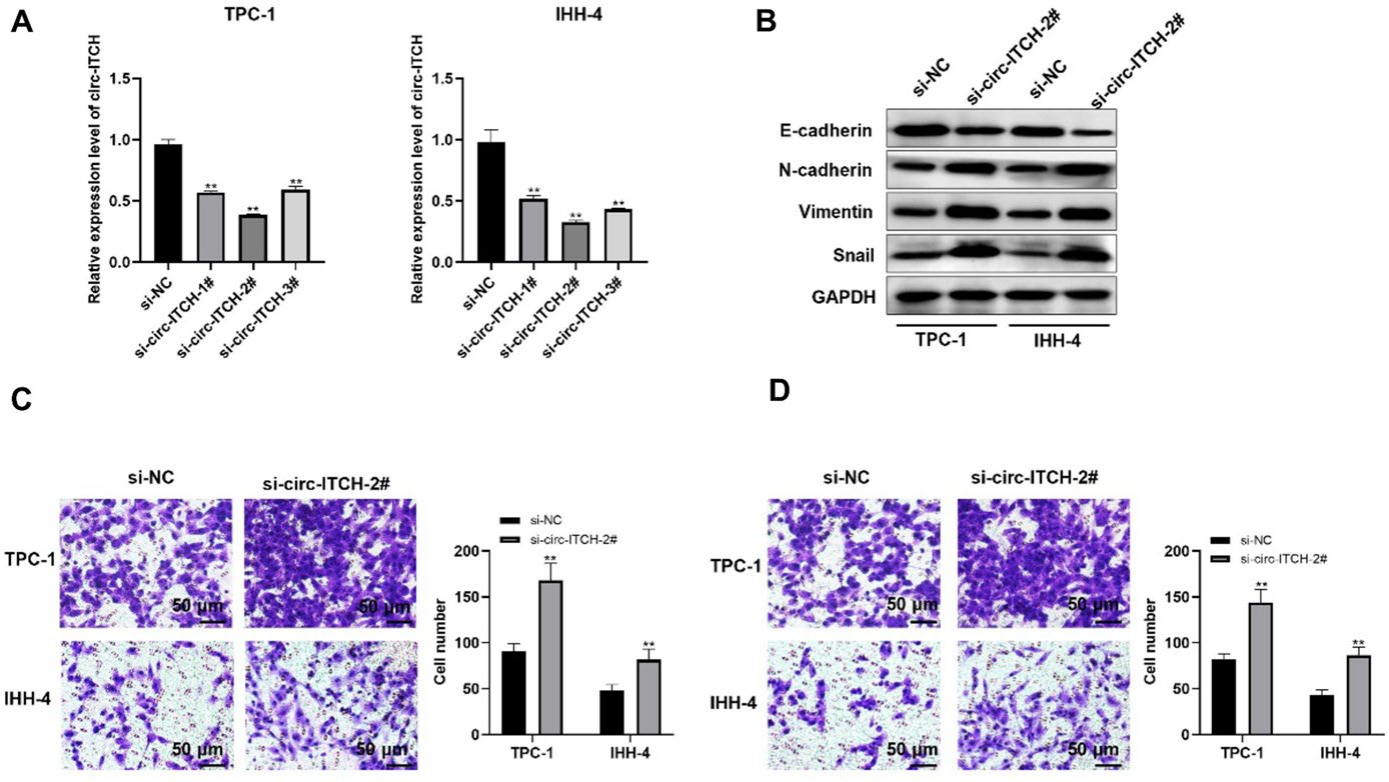
Circ-ITCH knockdown promotes PTC cells progression. **A** The expression level of circ-ITCH (*p* < 0.05). **B** The level of proteins related to EMT in TPC-1 as well as IHH-4 cells. **C** and **D** The migration (**C**) and invasion (**D**) of cells (*p* < 0.05)

Next, RNA interference technology (si-RNA) was introduced to decreased the circ-ITCH expression in TPC-1 as well as IHH-4 cell lines, which is verified in Fig. 3A and si-circ-ITCH-2# exhibited the best efficiency (Fig. 3A). A series of functional experiments showed that silencing circ-ITCH-induced EMT facilitated migration in addition to invasion in PTC cells (Fig. 3B–D).

### MiR-106a-5p is the Direct Target of circ-ITCH

**Fig. 4:**
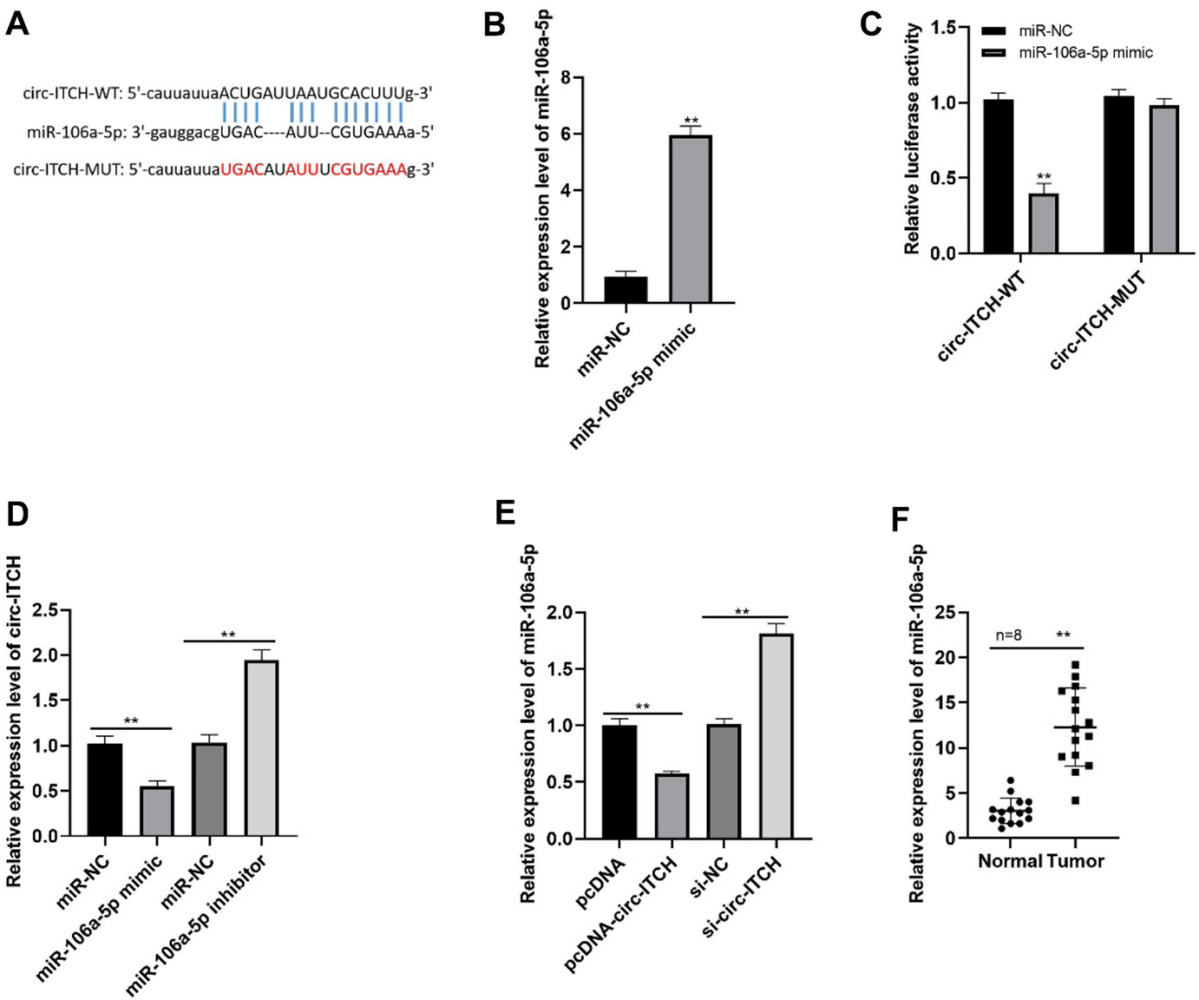
MiR-106a-5p is the direct target of circ-ITCH. **A** The binding sequence of miR-106a-5p and circ-ITCH. **B** The miR-106-5p level in cells (*p* < 0.05). **C** The luciferase activity (*p* < 0.05). **D** The circ-ITCH expression in cells (*p* < 0.05). **E** The miR-106-5p expression tested via qRT-PCR (*p* < 0.05). **F** The miR-106-5p expression in tissues (*p* < 0.05)

To uncover more detailed molecular mechanisms by which circ-ITCH exerts its role in PTC cells, we screened the Starbase database and discovered miR-106a-5p possessed the binding sites with circ-ITCH (Fig. 4A). Transfection of miR-106a-5p mimic in TPC-1 cells notably upregulated miR-106a-5p level (Fig. 4B). Besides, miR-106a-5p suppressed the luciferase production of circ-ITCH-WT (wild type) (Fig. 4C), while there is little effect on that of circ-ITCH-MUT (mutant) (Fig. 4C). In addition, miR-106a-5p mimic reduced circ-ITCH expression, whereas miR-106a-5p inhibitor increased it obviously (Fig. 4D). Moreover, as shown in Fig. 4E, circ-ITCH overexpression dramatically inhibited miR-106a-5p expression but circ-ITCH knockdown exerted opposite effects (Fig. 4E). Furthermore, miR-106a-5p was highly expressed in PTC tissues (Fig. 4F). The findings indicated that miR-106a-5p was negatively mediated via circ-ITCH.

### The Effect of circ-ITCH on PTC Cells Relies on miR-106a-5p

**Fig. 5:**
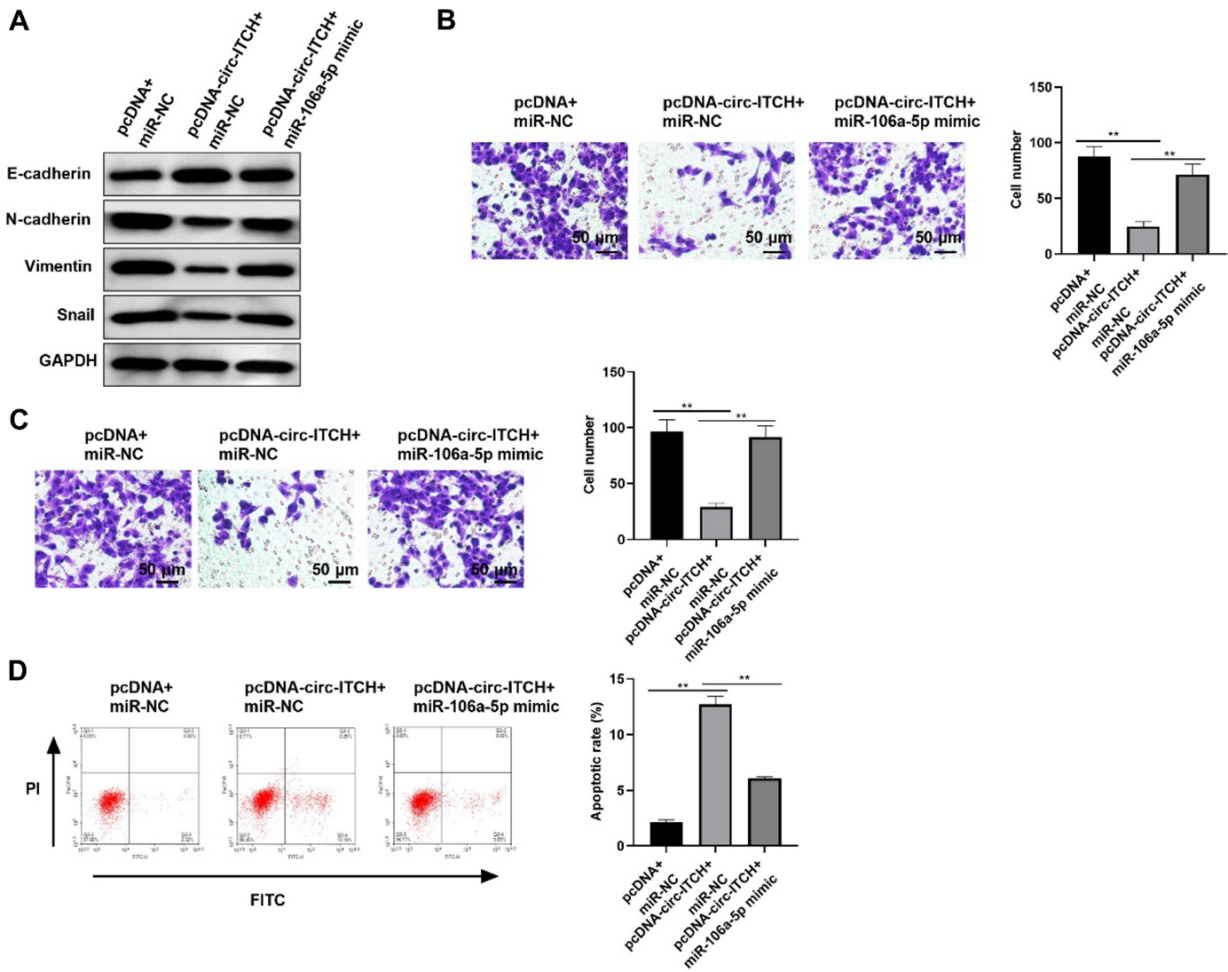
The effect of ITCH on PTC cells relies on miR-106a-5p. **A** The level of proteins related to EMT in cells. **B** and **C** The migration (**B**) as well as invasion (**C**) of TPC-1 cells (*p* < 0.05). **D** The apoptosis rate of cells (*p* < 0.05)

Subsequently, TPC-1 cells were co-transfected with pcDNA3.1-circ-ITCH or blank plasmid and miR-NC or miR-106a-5p mimic. Western blot assay illustrated that inhibitory EMT progression caused via circ-ITCH overexpression was eliminated through miR-106a-5p mimic (Fig. 5A). In addition, transwell assay also proved that the weakened effects of circ-ITCH overexpression on migration and invasion were partly counteracted by transfecting miR-106a-5p mimic (Fig. 5B, C). Furthermore, the increased apoptotic rate induced by pcDNA3.1-circ-ITCH was effectively reduced by miR-106a-5p overexpression (Fig. 5D). Collectively, these findings confirmed that circ-ITCH played its roles in PTC cells at least partly by mediating miR-106a-5p.

### Circ-ITCH Positively Regulates JAZF1

**Fig. 6:**
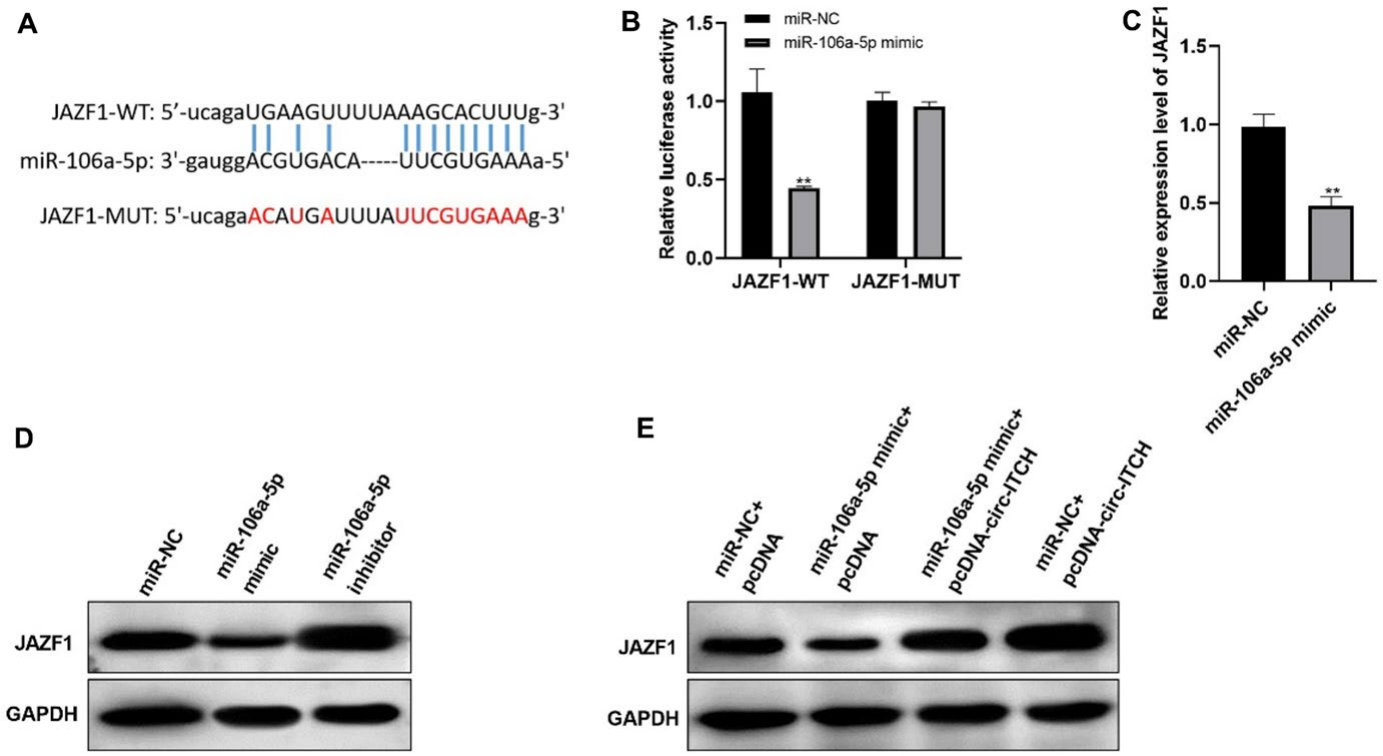
Circ-ITCH positively regulates JAZF1. **A** The binding sequence between miR-106a-5p and JAZF1. **B** The luciferase activity of circ-ITCH-WT and circ-ITCH-MUT (*p* < 0.05). **C**–**E** The mRNA (**C**) as well as protein (**D**, **E**) level of JAZF1 in TPC-1 cells (*p* < 0.05)

StarBase database predicted that numerous genes could be targeted through miR-106a-5p, among which JAZF1 caught our attention (Fig. 6A). It has reported that JAZF1 is closely associated with the development of PTC cells (Peng et al. 2017). Besides, luciferase activity and qRT-PCR assays proved miR-106a-5p mimic notably weakened the transcriptional activity of JAZF1 (Fig. 6B, C). Meanwhile, miR-106a-5p mimic reduced JAZF1 expression, indicating that JAZF1 was negatively regulated via miR-106a-5p (Fig. 6D). Given the relationship of miR-106a-5p and JAZF1, we hypothesized that circ-ITCH and JAZF1 competitively bind with miR-106a-5p in PTC cells. As expected, co-transfecting pcDNA3.1-circ-ITCH as well as miR-106a-5p mimic effectively recovered the expression level of JAZF1. Moreover, circ-ITCH overexpression alone increased JAZF1 expression obviously (Fig. 6E). These findings revealed that JAZF1 might be involved in the regulatory network of circ-ITCH/miR-106a-5p in PTC cells.

### Circ-ITCH Overexpression Suppresses PTC Cells Progression by Modulating JAZF1

**Fig. 7:**
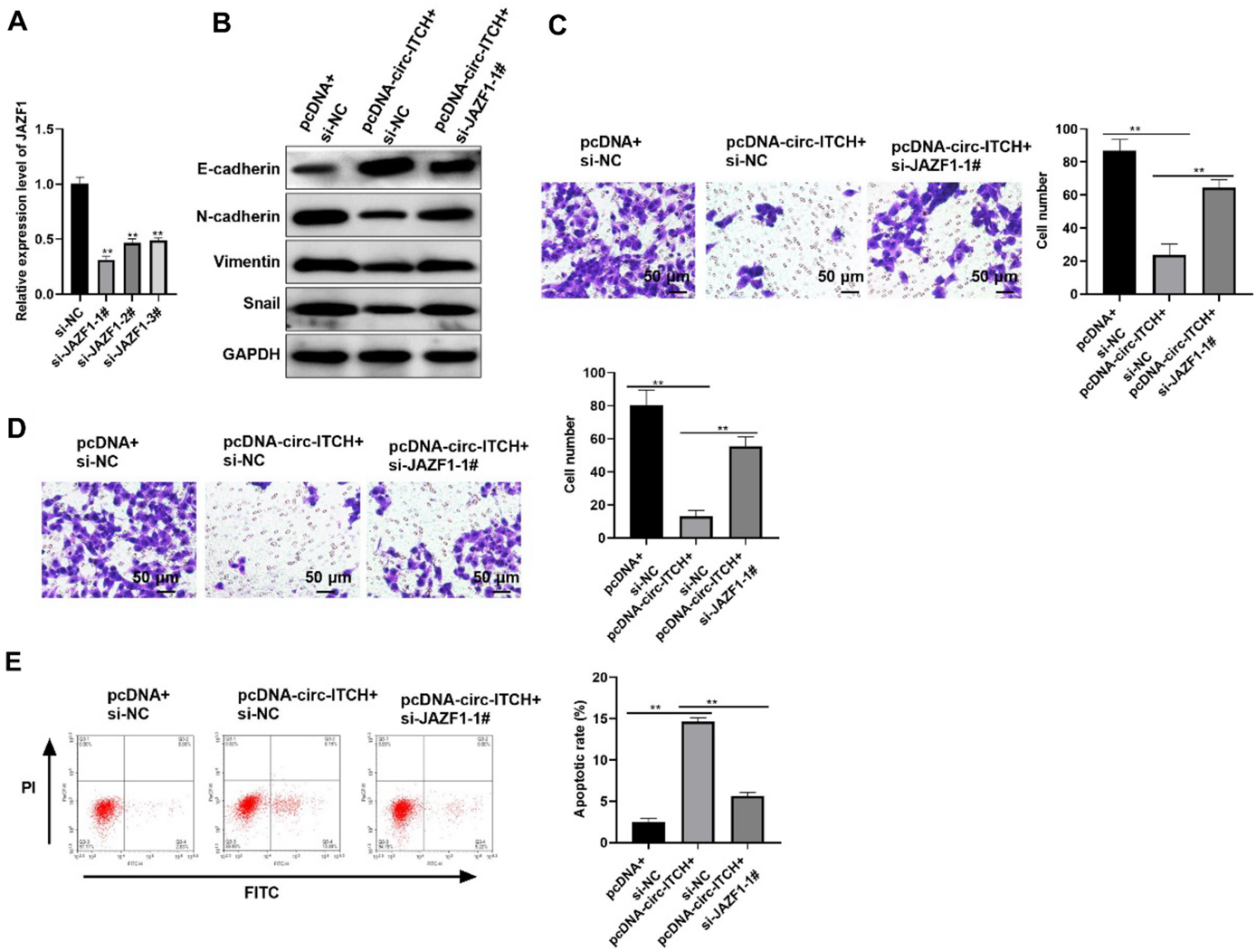
Circ-ITCH overexpression suppresses PTC cells progression by modulating JAZF1. **A** The mRNA level of JAZF1 in cells (*p* < 0.05). **B** The expression level of proteins related to EMT in cells. **C** and **D** The migration (**C**) as well as invasion (**D**) of cells (*p* < 0.05). **E** The apoptosis rate of cells

To investigate the role of JAZF1 in PTC cells progression influenced by circ-ITCH, JAZF1 was downregulated by RNA interference technology. As exhibited in Fig. 7A, JAZF1 expression was reduced by si-JAZF1-1#, si-JAZF1-2#, and si-JAZF1-3#, especially by si-JAZF1-1# (*p* < 0.05, Fig. 7A). We chose TPC-1-si-JAZF1-1# cell line for next explorations. Overexpression of circ-ITCH inhibited EMT, migration, as well as invasion, which was reversed via silencing JAZF1 (*p* < 0.05, Fig. 7B–D). Moreover, JAZF1 inhibition partly decreased apoptotic cells induced by circ-ITCH overexpression (Fig. 7E). These data proved that circ-ITCH suppressed progression and promotes apoptosis of PTC cells at least partly by modulating JAZF1, the target gene of miR-106a-5p.

## Discussion

CircRNAs are reported to participate in various biological progresses including tumor progression, and circRNAs might be serve as vital targets for treating diverse cancers (Zhang et al. 2018). Recent studies revealed that circRNAs are tightly related to the development of PTC. For instance, Circ-BACH2 exerts the essential effects on PTC via miR-139-5p/LMO4 network (Cai et al. 2019). Circ-FOXM1 modulating PTC development through miR-1179/HMGB1 pathway (Ye et al. 2020). Besides, circ-ITCH is reported to inhibit cell growth as well as invasion and facilitates apoptosis in PTC cells (Wang et al. 2018). Nevertheless, more mechanisms of circ-ITCH in PTC development need to be further investigated. Consistently, our present study confirmed circ-ITCH was under-expressed in PTC tissues in addition to cells. Additionally, circ-ITCH overexpression reduced PTC cells viability, migrated and invasive ability, and accelerated apoptosis, whereas circ-ITCH knockdown played opposite roles. Recent reporters have discovered that EMT is the main driving force for PTC metastasis (Shakib et al. 2019). EMT leads tumor cells to lose polarity as well as adhesion, thereby occurring invasion and metastasis (Gugnoni et al. 2017). To the best of our knowledge, this investigation revealed for the first time that circ-ITCH overexpression inhibited EMT process in PTC cells, while silencing circ-ITCH promoted it remarkably.

It is reported that circRNAs was able to exert its roles in PTC by interacting with miRNAs as the competitive endogenous RNA (ceRNA) (Chan and Tay 2018). For instance, Wang et al. demonstrated circ-ITCH could affect PTC progression through miR-22-3p/CBL/β-catenin signal (Wang et al. 2018). However, both miR-22-3p mimics and silencing CBL only partially recovered the effects of ITCH overexpression, suggesting that molecular network of circ-ITCH in the development of PTC is complicated and it might inhibit PTC progression by mediating other miRNAs as well as their target genes. In this investigation, StarBase database in addition to dual luciferase detection confirmed that miR-106a-5p was the target of circ-ITCH. MiR-106-5p is implicated in diverse tumorigenesis. For example, miR-106a5p/STAT3 axis is associated with the development and ferroptosis in breast cancer (Zhang et al. 2021). Besides, miR-106a-5p/PAK5 pathway reduced the migrated ability and invasive ability of renal cell carcinoma (Pan et al. 2017). Our present study firstly verified that circ-ITCH decreased the miR-106a-5p level in PTC cells. Moreover, the rescue assays demonstrated miR-106a-5p partially eliminated suppressive effects of circ-ITCH overexpression on EMT, thereby recovering migration as well as invasion of PTC cells. Thus, these findings suggested circ-ITCH exerted its roles in PTC cell at least partly via miR-106a-5p.

Increasing evidence reported that miRNAs could reduce the expression of target genes by degrading target mRNA or inhibiting its transcription (Garg et al. 2014). Here, we proved JAZF1 was the target of miR-106a-5p according to StarBase database and luciferase activity detection. JAZF1, a newly identified repressor of transforming growth factor beta-activated kinase1 (TAK1), has been found to exert crucial roles in diabetes as well as lipid metabolism (Wei et al. 2018). Huang et al. discovered that JAZF1 is a tumor suppressor in PTC, while its upstream regulatory genes have not been defined. This work revealed miR-106a-5p inhibited JAZF1 expression in PTC cells, and the suppressive effect was effectively recovered by circ-ITCH overexpression. Moreover, silencing JAZF1 partly reversed EMT, migration, invasion, as well as the apoptosis of PTC cells affected by circ-ITCH overexpression, which indicated that circ-ITCH played its roles in PTC cells by mediating miR-106a-5p/JAZF1 axis.

There exist some limitations in the work. For instance, more samples are needed to verify whether circ-ITCH affects the prognosis of patients with PTC. Furthermore, the clinical functions of circ-ITCH will be investigated by employing a series of experiments.

## Conclusion

This investigation firstly confirmed that circ-ITCH inhibited EMT, migration, as well as invasion and promoted apoptosis in PTC cells at least partly by modulating miR-106a-5p/JAZF1 axis. These findings provide a novel insight for treating PTC.

## Data Availability

The data used to support these findings of this study are included in this study.

## References

[CR1] Cai X, Zhao Z, Dong J, Lv Q, Yun B, Liu J, Shen Y, Kang J, Li J (2019) Circular RNA circBACH2 plays a role in papillary thyroid carcinoma by sponging miR-139-5p and regulating LMO4 expression. Cell Death Dis 10:184 30796202 10.1038/s41419-019-1439-y PMC6385235

[CR2] Chan JJ, Tay Y (2018) Noncoding RNA:RNA regulatory networks in cancer. Int J Mol Sci 19:1310 29702599 10.3390/ijms19051310 PMC5983611

[CR3] Chen AY, Jemal A, Ward EM (2009) Increasing incidence of differentiated thyroid cancer in the United States, 1988–2005. Cancer 115:3801–3807 19598221 10.1002/cncr.24416

[CR4] Cheng CJ, Bahal R, Babar IA, Pincus Z, Barrera F, Liu C, Svoronos A, Braddock DT, Glazer PM, Engelman DM, Saltzman WM, Slack FJ (2015) MicroRNA silencing for cancer therapy targeted to the tumour microenvironment. Nature 518:107–110 25409146 10.1038/nature13905 PMC4367962

[CR5] Frohlich E, Wahl R (2014) The current role of targeted therapies to induce radioiodine uptake in thyroid cancer. Cancer Treat Rev 40:665–674 24485648 10.1016/j.ctrv.2014.01.002

[CR6] Garg M, Kanojia D, Okamoto R, Jain S, Madan V, Chien W, Sampath A, Ding LW, Xuan M, Said JW, Doan NB, Liu LZ, Yang H, Gery S, Braunstein GD, Koeffler HP (2014) Laminin-5γ-2 (LAMC2) is highly expressed in anaplastic thyroid carcinoma and is associated with tumor progression, migration, and invasion by modulating signaling of EGFR. J Clin Endocrinol Metab 99:E62-72 24170107 10.1210/jc.2013-2994 PMC3879679

[CR7] Garg M, Kanojia D, Mayakonda A, Ganesan TS, Sadhanandhan B, Suresh S, Nagare RP, Said JW, Doan NB, Ding LW, Baloglu E, Shacham S, Kauffman M, Koeffler HP (2017) Selinexor (KPT-330) has antitumor activity against anaplastic thyroid carcinoma in vitro and in vivo and enhances sensitivity to doxorubicin. Sci Rep 7:9749 28852098 10.1038/s41598-017-10325-x PMC5575339

[CR8] Gugnoni M, Sancisi V, Gandolfi G, Manzotti G, Ragazzi M, Giordano D, Tamagnini I, Tigano M, Frasoldati A, Piana S, Ciarrocchi A (2017) Cadherin-6 promotes EMT and cancer metastasis by restraining autophagy. Oncogene 36:667–677 27375021 10.1038/onc.2016.237

[CR9] Huang L, Cai Y, Luo Y, Xiong D, Hou Z, Lv J, Zeng F, Yang Y, Cheng X (2019) JAZF1 suppresses papillary thyroid carcinoma cell proliferation and facilitates apoptosis via regulating TAK1/NF-κB pathways. Onco Targets Ther 12:10501–10514 31819531 10.2147/OTT.S230597 PMC6897071

[CR10] Johnson JA, Watson JK, Nikolić MZ, Rawlins EL (2018) Fank1 and Jazf1 promote multiciliated cell differentiation in the mouse airway epithelium. Biology open 7:bio033944 29661797 10.1242/bio.033944 PMC5936064

[CR11] Kloosterman WP, Plasterk RH (2006) The diverse functions of microRNAs in animal development and disease. Dev Cell 11:441–450 17011485 10.1016/j.devcel.2006.09.009

[CR12] Lim H, Devesa SS, Sosa JA, Check D, Kitahara CM (2017) Trends in thyroid cancer incidence and mortality in the United States, 1974–2013. JAMA 317:1338–1348 28362912 10.1001/jama.2017.2719 PMC8216772

[CR13] Liu Y, Chen S, Zong ZH, Guan X, Zhao Y (2020) CircRNA WHSC1 targets the miR-646/NPM1 pathway to promote the development of endometrial cancer. J Cell Mol Med 24:6898–6907 32378344 10.1111/jcmm.15346 PMC7299690

[CR14] Liu T, Yang C, Wang W, Liu C (2021) LncRNA SGMS1-AS1 regulates lung adenocarcinoma cell proliferation, migration, invasion, and EMT progression via miR-106a-5p/MYLI9 axis. Thorac Cancer 12:2104–2112 34061466 10.1111/1759-7714.14043 PMC8287014

[CR15] Ma J, Wang W, Azhati B, Wang Y, Tusong H (2020) miR-106a-5p functions as a tumor suppressor by targeting VEGFA in renal cell carcinoma. Dis Markers 2020:8837941 33224312 10.1155/2020/8837941 PMC7669356

[CR16] Martello G, Rosato A, Ferrari F, Manfrin A, Cordenonsi M, Dupont S, Enzo E, Guzzardo V, Rondina M, Spruce T, Parenti AR, Daidone MG, Bicciato S, Piccolo S (2010) A MicroRNA targeting dicer for metastasis control. Cell 141:1195–1207 20603000 10.1016/j.cell.2010.05.017

[CR17] Memczak S, Jens M, Elefsinioti A, Torti F, Krueger J, Rybak A, Maier L, Mackowiak SD, Gregersen LH, Munschauer M, Loewer A, Ziebold U, Landthaler M, Kocks C, le Noble F, Rajewsky N (2013) Circular RNAs are a large class of animal RNAs with regulatory potency. Nature 495:333–338 23446348 10.1038/nature11928

[CR18] Meng F, Lin Y, Yang M, Li M, Yang G, Hao P, Li L (2018) JAZF1 inhibits adipose tissue macrophages and adipose tissue inflammation in diet-induced diabetic mice. Biomed Res Int 2018:4507659 29765984 10.1155/2018/4507659 PMC5885486

[CR19] Nakajima T, Fujino S, Nakanishi G, Kim YS, Jetten AM (2004) TIP27: a novel repressor of the nuclear orphan receptor TAK1/TR4. Nucleic Acids Res 32:4194–4204 15302918 10.1093/nar/gkh741 PMC514368

[CR20] Pan YJ, Wei LL, Wu XJ, Huo FC, Mou J, Pei DS (2017) MiR-106a-5p inhibits the cell migration and invasion of renal cell carcinoma through targeting PAK5. Cell Death Dis 8:e3155 29072688 10.1038/cddis.2017.561 PMC5680926

[CR21] Peng N, Shi L, Zhang Q, Hu Y, Wang N, Ye H (2017) Microarray profiling of circular RNAs in human papillary thyroid carcinoma. PLoS ONE 12:e0170287 28288173 10.1371/journal.pone.0170287 PMC5347999

[CR22] Regenstein M, Nocella K, Jewers MM, Mullan F (2016) The cost of residency training in teaching health centers. N Engl J Med 375:612–614 27376580 10.1056/NEJMp1607866

[CR23] Ren C, Liu J, Zheng B, Yan P, Sun Y, Yue B (2019) The circular RNA circ-ITCH acts as a tumour suppressor in osteosarcoma via regulating miR-22. Artif Cells Nanomed Biotechnol 47:3359–3367 31387405 10.1080/21691401.2019.1649273

[CR24] Schneider DF, Chen H (2013) New developments in the diagnosis and treatment of thyroid cancer. Cancer J Clin 63:374–394 10.3322/caac.21195 PMC3800231 23797834

[CR25] Shakib H, Rajabi S, Dehghan MH, Mashayekhi FJ, Safari-Alighiarloo N, Hedayati M (2019) Epithelial-to-mesenchymal transition in thyroid cancer: a comprehensive review. Endocrine 66:435–455 31378850 10.1007/s12020-019-02030-8

[CR26] Shang D, Liu Y, Zhang J, Hu X (2020) Peroxisome proliferator-activated receptor γ (PPARγ) suppresses the proliferation and metastasis of patients with urothelial carcinoma after renal transplantation by inhibiting LEF1/β-catenin signaling. Bioengineered 11:1350–1367 33289586 10.1080/21655979.2020.1843834 PMC8291807

[CR27] Tang D, Geng L, Ma J (2021) lncRNA PROX1-AS1 mediates the migration and invasion of placental trophoblast cells via the miR-211-5p/caspase-9 axis. Bioengineered 12:4100–4110 34288800 10.1080/21655979.2021.1953213 PMC8806442

[CR28] Wang M, Chen B, Ru Z, Cong L (2018) CircRNA circ-ITCH suppresses papillary thyroid cancer progression through miR-22-3p/CBL/β-catenin pathway. Biochem Biophys Res Commun 504:283–288 30190130 10.1016/j.bbrc.2018.08.175

[CR29] Wang X, Chen Y, Dong K, Ma Y, Jin Q, Yin S, Zhu X, Wang S (2021) Effects of FER1L4-miR-106a-5p/miR-372-5p-E2F1 regulatory axis on drug resistance in liver cancer chemotherapy. Mol Ther Nucleic Acids 24:449–461 33868788 10.1016/j.omtn.2021.02.006 PMC8040129

[CR30] Wei Q, Zhou B, Yang G, Hu W, Zhang L, Liu R, Li M, Wang K, Gu HF, Guan Y, Zhu Z, Zheng H, Peng J, Li L (2018) JAZF1 ameliorates age and diet-associated hepatic steatosis through SREBP-1c -dependent mechanism. Cell Death Dis 9:859 30154417 10.1038/s41419-018-0923-0 PMC6113258

[CR31] Yang C, Yuan W, Yang X, Li P, Wang J, Han J, Tao J, Li P, Yang H, Lv Q, Zhang W (2018) Circular RNA circ-ITCH inhibits bladder cancer progression by sponging miR-17/miR-224 and regulating p21. PTEN Expr Mol Cancer 17:19 10.1186/s12943-018-0771-7 PMC5793418 29386015

[CR32] Ye M, Hou H, Shen M, Dong S, Zhang T (2020) Circular RNA circFOXM1 plays a role in papillary thyroid carcinoma by sponging miR-1179 and regulating HMGB1 expression. Mol Ther Nucleic Acids 19:741–750 31951855 10.1016/j.omtn.2019.12.014 PMC6965511

[CR33] Zhang HD, Jiang LH, Sun DW, Hou JC, Ji ZL (2018) CircRNA: a novel type of biomarker for cancer. Breast Cancer 25:1–7 28721656 10.1007/s12282-017-0793-9

[CR34] Zhang H, Ge Z, Wang Z, Gao Y, Wang Y, Qu X (2021) Circular RNA RHOT1 promotes progression and inhibits ferroptosis via mir-106a-5p/STAT3 axis in breast cancer. Aging 13:8115–8126 33686957 10.18632/aging.202608 PMC8034942

[CR35] Zhao L, Ma N, Liu G, Mao N, Chen F, Li J (2021) Lidocaine inhibits hepatocellular carcinoma development by modulating circ_ITCH/miR-421/CPEB3 axis. Dig Dis Sci 66:4384–4397 33433806 10.1007/s10620-020-06787-1

[CR36] Zheng YJ, Zhao JY, Liang TS, Wang P, Wang J, Yang DK, Liu ZS (2019) Long noncoding RNA SMAD5-AS1 acts as a microRNA-106a-5p sponge to promote epithelial mesenchymal transition in nasopharyngeal carcinoma. FASEB J 33:12915–12928 31557058 10.1096/fj.201900803R PMC6902713

[CR37] Zhou R, Wu Y, Wang W, Su W, Liu Y, Wang Y, Fan C, Li X, Li G, Li Y, Xiong W, Zeng Z (2018) Circular RNAs (circRNAs) in cancer. Cancer Lett 425:134–142 29625140 10.1016/j.canlet.2018.03.035

[CR38] Zhu X, Du J, Gu Z (2020) Circ-PVT1/miR-106a-5p/HK2 axis regulates cell growth, metastasis and glycolytic metabolism of oral squamous cell carcinoma. Mol Cell Biochem 474:147–158 32737775 10.1007/s11010-020-03840-5

